# Early life factors in relation to albuminuria and estimated glomerular filtration rate based on cystatin C and creatinine in adults from a Swedish population-based cohort study

**DOI:** 10.1007/s40620-021-01159-y

**Published:** 2021-10-08

**Authors:** Agne Laucyte-Cibulskiene, Shantanu Sharma, Anders Christensson, Peter M. Nilsson

**Affiliations:** 1 Department of Clinical Sciences, Skane University Hospital, Lund University, Ruth Lundskogs gata 14, 205 02 Malmö, Sweden; 2 Department of Nephrology, Skane University Hospital, Lund University, 205 02 Malmö, Sweden

**Keywords:** Albuminuria, Birth weight, Cystatin C, Early life, Epidemiology, Estimated glomerular filtration rate

## Abstract

**Background:**

Early life factors influence the number of nephrons a person starts life with and a consequence of that is believed to be premature kidney ageing. Thus, we aimed to identify early life factors associated with cystatin C and creatinine-based estimated glomerular filtration (eGFR) rate equations and urine -albumin-to-creatinine ratio after a follow-up of 46–67 years.

**Methods:**

The study included 593 Swedish subjects without diabetes mellitus from the Malmo Diet Cancer Cohort. Perinatal data records including birth weight, gestational age, placenta weight and maternal related risk factors were analysed. eGFR was determined by Chronic Kidney Disease Epidemiology (CKD-EPI), the Lund-Malmö revised and Caucasian, Asian, Paediatric, and Adult (CAPA) equations. Postnatal growth phenotypes were defined as low (≤ 0) or high (> 0) birth weight z-score, or low (≤ median) or high (> median) body mass index at 20 years of age.

**Results:**

In women, lower birth weight was associated with lower eGFR (CAPA; CKD-EPI cystatin C). Birth weight z-score predicted adult albuminuria specifically in men (OR 0.75, 95% CI [0.58; 0.96]). Women with high birth weight z-score and low BMI at 20 years had lower eGFR (CAPA; CKD-EPI cystatin C; *p* = 0.04). Men with high birth weight z-score and high BMI at 20 years had lower risk for albuminuria (OR 0.35, 95% CI [0.12; 0.93]).

**Conclusions:**

Lower birth weight, prematurity and postnatal growth curve have a potential sex- specific effect of early exposure to an adverse environment on lower cystatin C-based eGFR and albuminuria later in life. Cystatin C compared to creatinine -eGFR equations shows a higher ability to detect these findings.

**Graphic abstract:**

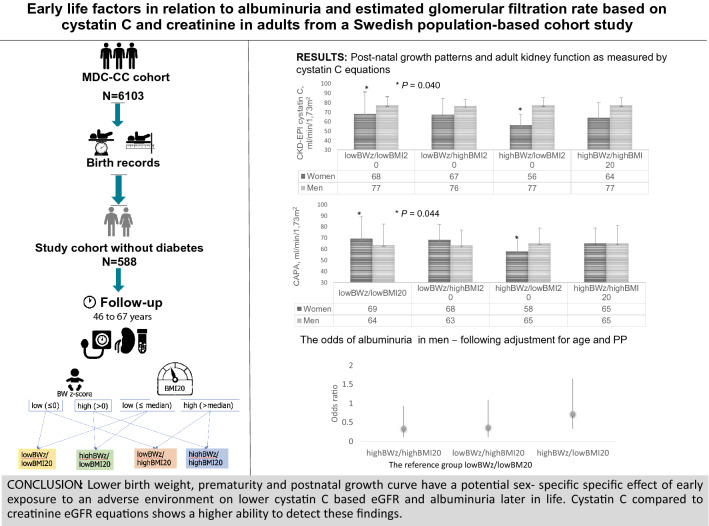

## Introduction

### Background

Kidney senescence, dependent on predicted anatomical and structural changes, is a separate process from kidney disease [1] and manifests with declining nephron number and size. Aged-induced glomerulosclerosis is followed by compensatory glomerular hypertrophy, glomerular basement membrane (GBM) thickening, tubular atrophy, interstitial fibrosis, and arteriosclerosis [2]. Normal kidney ageing and chronic kidney disease (CKD) share similar pathophysiological [2] and maladaptive repair [3] pathways despite different causality. CKD is very common in the general population and as many as 10% of individuals suffer from this neglected “non-communicable disease” associated with higher mortality [4]. Among CKD risk factors, e.g. ageing, gender, family history, obesity, smoking, hypertension, diabetes mellitus, etc. [5], early life factors also play an important role in CKD progression and renal outcome prediction [6, 7].

Renal functional capacity is influenced by factors acting early in life, such as intrauterine environment, maturity, birth weight, length at birth, placental weight, etc. [7–9]. Early life factors influence the number of nephrons a person starts with at birth, and the consequence of low nephron number is believed to be premature kidney ageing, hypertension and CKD later in one’s lifetime [10]. There is a debate on whether low birth weight newborns have low podocyte endowment [11] which increases the risk of renal pathology. In utero perturbation leads to altered kidney development that affects low birth weight and/or diminished nephron number causing decreased filtration surface area/altered sodium handling and eventually leading to increased programmed risk of adult hypertension [12] and kidney diseases [13].

In a recent meta-analysis [7] the strong relationship between low birth weight, or small for gestational age, and renal dysfunction in adulthood was observed. Another longitudinal result came from the Helsinki Birth Cohort study [8] which found that smaller body size at birth influences the onset of CKD, although mainly in men. There are other definitive triggers for the development of CKD such as prematurity [9] and preeclampsia [14]. On the contrary, the role of both high birth weight, a proven risk marker for the metabolic syndrome, and maternal obesity [15] has to be elucidated for kidney health.

The evaluation of kidney function is based on glomerular filtration rate (GFR) that can be measured (mGFR) or estimated (eGFR) from equations based on creatinine or cystatin C which are used worldwide [16]. During previous decades it was shown that cystatin C is superior to creatinine in estimating kidney function [17]. Notably, most reports addressing early life factors in the context of adult kidney function use creatinine-based eGFR equations and/or albuminuria but lack longer follow-up (> 30 years) [7, 18].

### Objectives

We aimed to identify how early life factors affect adult kidney function, by introducing cystatin C-based GFR equations and kidney damage, determined by urine albumin-to-creatinine ratio (UACR), in a cohort with very long follow-up, i.e., more than 40 years. We hypothesize that in middle-aged and elderly Swedish subjects, changes in kidney function capacity, represented as lower eGFR, and albuminuria, are influenced by early life factors such as low birth weight, prematurity, discrepant birth weight to placental weight ratio, and postnatal growth mismatch phenotype.

## Methods

### Study design and settings

The observational cohort study was performed with subjects that participated in the population-based Malmö Diet and Cancer Study (MDCS) [19] between 1991 and 1996. MDCS comprises 17,035 women and 11,063 men born between 1923 and 1950 and residing in Malmö*.* Furthermore, a random 50% of MDCS participants were invited to participate in carotid artery disease epidemiological analysis (the cardiovascular arm of MDCS (MDCS‐CC)). Due to lower response rate a total of 6,103 participants completed the MDCS-CC study [20, 21].

### Participants

In this study, MDCS-CC participants without diabetes mellitus for whom perinatal data records were available including birth weight, birth length, head circumference, gestational age, placenta weight, and maternal risk factors at baseline (year 1926–1945), and cystatin C and creatinine measurements after the mean follow-up time of 58 ± 5 years (range: 46 to 67 years) (96 women and 497 men) were included. For more detailed participant recruitment and data collection see Gerdtsson et al. [22] and Lahmann et al*.* [23]. The mean follow-up time was 58 ± 5 years (range 46–67 years).

### Measurements

#### Early life factors

Birth records were collected from archives at local and regional hospitals and were located using methods as described elsewhere [22, 23]. Birth records include data regarding birth weight (grams), length at birth (centimetres), head circumference (centimetres), placental weight (grams), and last menstrual period (day). Gestational age at delivery was calculated from last menstruation period until the birth week, prematurity was defined as being born alive before 37 weeks of pregnancy. Birth weight to placenta weight ratio was calculated as birth weight (g)/placenta weight (g).

#### Postnatal growth phenotype

The *mismatch* term defines pre- and postnatal growth patterns influencing body weight at 20 years of age. To test different postnatal growth curves, or *mismatch* phenotypes, we divided all participants into four subgroups by combining sex-specific low (≤ 0) or high (> 0) birth weight z-score (*low-birth weight-z-score* or *high-birth weight-z-score*, respectively) with low (≤ median) or high (> median) recalled body mass index (BMI) at 20 years of age (*lowBMI20* or *highBMI20*, respectively). These four subgroups were as follows: *low-birth weight-z-score/low BMI20*, *low-birth weight-z-score/high BMI20* (the *mismatch* or postnatal catch-up growth phenotype), *high-birth weight-z-score/low BMI20*, and *high-birth weight-z-score/high BMI20*.

Birth weight z-scores (gender-specific birth weight z-score and combined birth weight z-score) were acquired by using equation from official Swedish growth-charts as reported by Marsal et al. [24]. BMI20 calculation: self-reported body weight (kg) at 20 years of age divided by squared height (m).

#### Follow-up measurements

All participants underwent anthropometric measurements (weight (kilograms), height (centimetres)) and systolic and diastolic blood pressure (SBP and DBP, mmHg) measurements obtained by trained nurses between 1991 and 1996. Pulse pressure (PP) was calculated as follows: SBP-DBP (mmHg). Questions concerning self-reported body weight at 20 years of age were included in a self-administrated questionnaire [25]. Fasting blood samples were drawn and immediately frozen to − 80 °C and stored in a biobank as reported elsewhere [26]. Plasma creatinine (μmol/L) concentration was analysed by the Jaffé method [27] and using a calibrator traceable to the international standardization with isotope dilution mass spectrometry (IDMS) [28]. Plasma cystatin C (mg/L) was measured with a particle-enhanced immunonephelometric assay (N Latex Cystatin; Dade Behring, Deerfield, IL, USA). The values for cystatin C were analysed before the introduction of the world calibrator in 2010 and thus not standardized [29]. The reference values for the method were 0.53–0.95 mg/L. In order to standardize cystatin C values, we used cystatin C and creatinine within 2.5–97.5th percentile in a reference Swedish cohort and values in the same range in MDC-CC. Furthermore, sex-divided linear regression models with ln(cystatin c) as the dependent variable adjusted for age and for ln(creatinine) were created both in the reference cohort and in MDC-CC, and predicted cystatin C concentrations were acquired. Corrected cystatin C values were calculated by using the formula below:

$$ Corrected\;Cys\;C = \;Cys\;C_{MDS - CC} + predicted\;Cys\;C_{reference} - predicted\;Cys\;C_{MDS - CC} $$

Cys C—cystatin C, *reference*—the reference cohort.

### Kidney function evaluation

Estimated glomerular filtration rate was calculated according to 4 different equations: Chronic Kidney Disease Epidemiology Collaboration (CKD-EPI) 2012 creatinine and cystatin C equation  [30] (CKD-EPI creatinine, CKD-EPI cystatin C), cystatin C eGFR equation based on Caucasian, Asian, Paediatric, and Adult cohorts [31], the Lund-Malmö revised creatinine based eGFR equation (LMrev) [32].

Urine albumin-to-creatinine ratio (mg/mmol) was measured in morning urine samples as reported elsewhere [33] and albuminuria was defined as UACR ⩾3 mg/mmol. it is worth noting that 11% (*n* = 65) of participants had microalbuminuria and 2% (*n *= 11) had macroalbuminuria.

### Ethics

Each investigator committed to comply with legislation and to conduct the study in line with regulations, in accordance with the 1975 Declaration of Helsinki and its later amendments. The following ethical permissions apply for the cohort: MDCS (LU 51/90, 532/2006).

### Statistical analysis

All statistical analyses were conducted with Rcrmdr version R 3.6.2 GUI 1.70 El Capitan build macOS.

Continuous variables are expressed as mean ± standard deviations (SD) in parentheses, discrete variables as medians with min–max values in parentheses, and categorical variables as percentages with numbers in parentheses. Where appropriate, the equality of two populations for normally distributed continuous data were tested by an *F* test, and only after this did we perform a Student’s *t* test. Nonparametric tests, such as a two-sample Wilcoxon test, was performed on not normally distributed data. A Chi-square test was applied to categorical variables. To determine the correlation between two variables, a Pearson correlation test was performed and depicted as scatterplots. The ANOVA test was applied for comparison of age-adjusted eGFR equations in different postnatal growth phenotypes.

Multivariable linear regression analyses with creatinine and cystatin C equations as dependent variables were performed to identify early life factors independently associated with kidney function. UACR was categorized in two groups: normal UACR if < 3 mg/mmol, albuminuria if ⩾3 mg/mmol. Logistic regression analysis identified early life related variables as risk factors for albuminuria. A *p* value less than 0.05 was considered significant.

## Results

### Participants

**Fig.1:**
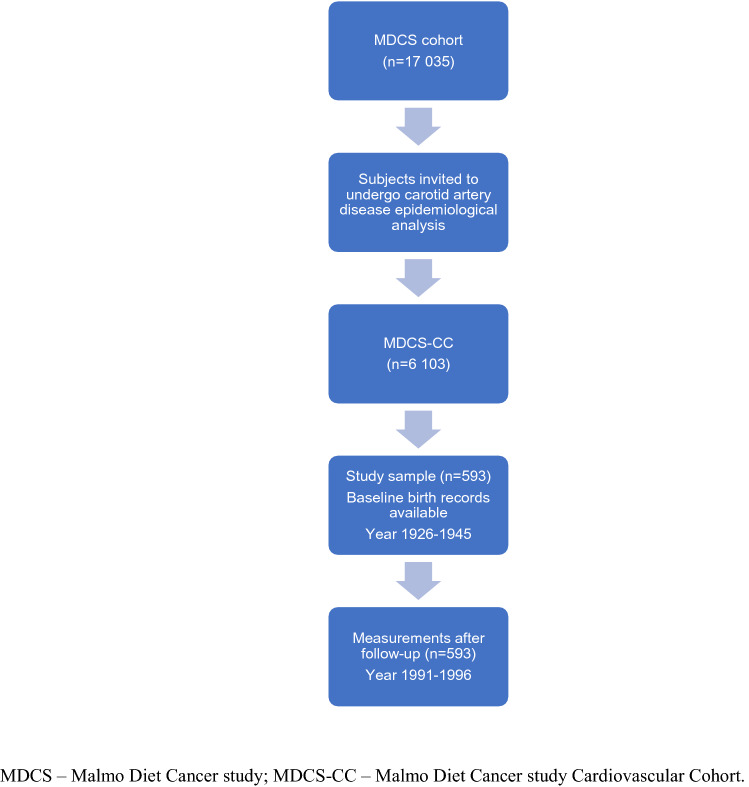
Flow chart of study sample. *MDCS* Malmo Diet Cancer study, *MDCS-CC* Malmo Diet Cancer study Cardiovascular Cohort

The study sample selection flow chart is presented in Fig. 1.

### General characteristics of the study population

General characteristics for men and women including the early life variables at birth and variables evaluated after a follow-up of 46–67 years are presented in Table 1. eGFR calculated by the CAPA equation and UACR were similar in both sexes. Birth weight percentiles (P) were as follows: in women P_2_—2,408 g, P_5_—2,550 g, P_10_—2,747.5 g, P_25_—3,045 g, P_50_—3,410 g, P_75_—3,762.5 g, P_90_—4,055 g, P_95_—4,388.75 g, P_98_—4,561 g; in men P_2_—2,233.6 g, P_5_—2,650 g, P_10_—2,850 g, P_25_—3,200 g, P_50_—3,550 g, P_75_—3,900 g, P_90_—4,200 g, P_95_ 4,352 g, P_98_—4,630.4 g.

**Table 1:** Study population—clinical characteristics and descriptive analyses

	Women (*n* = 96)	Men (*n* = 497)	*P*
Age, years	56 (5)	58 (5)	< 0.001
Body weight, kg	70 (13)	82 (12)	< 0.001
Height, cm	165 (6)	177 (7)	< 0.001
Body mass index, kg/m^2^	25.6 (4.4)	26.1 (3.3)	0.343
Systolic blood pressure, mmHg	136 (18)	144 (18)	< 0.001
Diastolic blood pressure, mmHg	84 (9)	89 (9)	< 0.001
Pulse pressure, mmHg	53 (14)	55 (14)	0.096
Gestational age, weeks	39 (2)	40 (2)	0.052
Head circumference, cm	35 (2)	35 (2)	0.965
Birth weight, g	3,396 (570)	3,534 (545)	0.032
Placenta weight, g	624 (124)	631 (138)	0.716
Birth weight to placenta weight ratio	5.5 (0.8)	5.7 (1.0)	0.070
Expected weight [23] (adjusted for gestational age), g	3,477 (374)	3,550 (392)	0.090
Expected weight [23] (adjusted for gestational age, sex), g	3,417 (354)	3,606 (415)	< 0.001
Cystatin C, mg/L	1.2 (0.2)	1.2 (0.2)	0.684
Creatinine, μmol/L	80 (12)	93 (14)	< 0.001
CAPA [31]	64 (16)	65 (16)	0.858
LMrev [32]	81 (12)	70 (11)	< 0.001
CKD-EPI cystatin C [30]	63 (17)	77 (8)	< 0.001
CKD-EPI creatinine [30]	73 (13)	79 (13)	< 0.001
Urine albumin-to-creatinine ratio, mg/mmol	2.1 (5.4)	3.1 (14.3)	0.504

Data expressed as Mean (± SD), and statistical comparisons by Student’s *t* test

*eGFR* estimated glomerular filtration rate, *CAPA*cystatin C eGFR equation based on Caucasian, Asian, Paediatric, and Adult cohorts, *LMrev* the Lund-Malmö revised creatinine based eGFR equation, *CKD-EPI cystatin C* the Chronic Kidney Disease Epidemiology Collaboration cystatin C equation, *CKD-EPI creatinine* the Chronic Kidney Disease Epidemiology Collaboration creatinine equation

### Early life factors and adult kidney function

**Fig.2:**
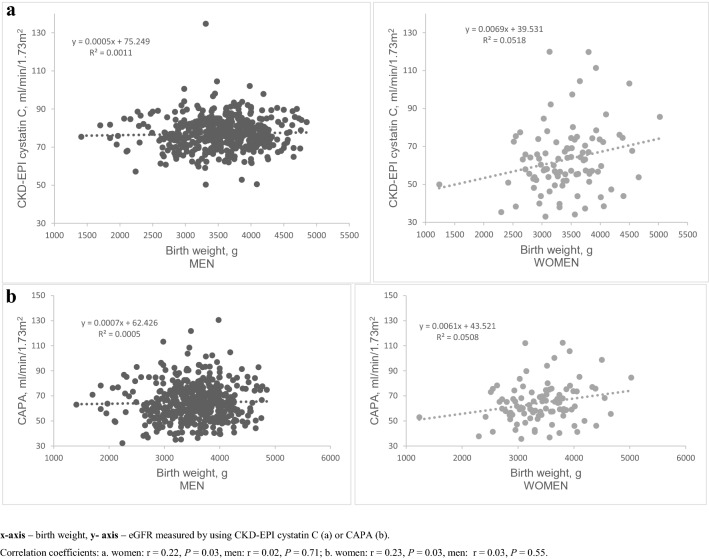
Birth weight correlation with adult kidney function measured by cystatin C equations in men and women. *x* axis—birth weight, *y* axis—eGFR measured by using CKD-EPI cystatin C (**a**) or CAPA (**b**). Correlation coefficients: **a** women: *r* = 0.22, *P* = 0.03, men: *r* = 0.02, *P* = 0.71; **b** women: *r* = 0.23, *P* = 0.03, men: *r* = 0.03, *P* = 0.55

**Fig.3:**
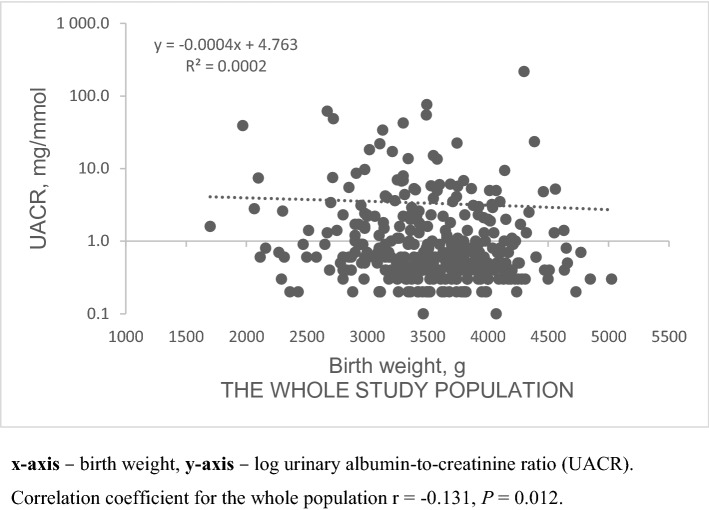
Urine albumin-to-creatinine ratio correlation with birth weight both in men and women. *x* axis—birth weight, *y* axis—log urinary albumin-to-creatinine ratio (UACR). Correlation coefficient for the whole population *r* = − 0.131, *P* = 0.012

Birth weight in women was positively correlated with adult kidney function measured by both cystatin C equations (CAPA and CKD-EPI cystatin C) (Fig. 2). However, there was no association between birth weight and adult kidney function in men for either cystatin C or creatinine equations. The correlations between early life factors, e.g. lower birth weight, and elevated UACR observed in the whole cohort were not sex-specific (Fig. 3).

### Linear regression analysis with eGFR as dependent variable

**Fig.4:**
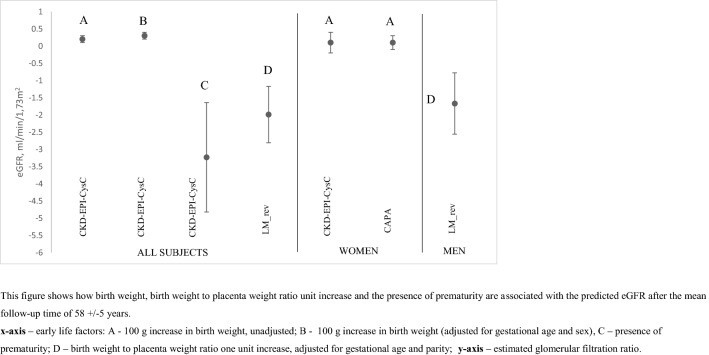
Early life factors as determinants of adult kidney function, measured by cystatin C or creatinine eGFR equations—following adjustments for age and pulse pressure. Depicting of multiple regression analysis. This figure shows how birth weight, birth weight to placenta weight ratio unit increase and the presence of prematurity are associated with the predicted eGFR after the mean follow-up time of 58 ± 5 years. *x* axis—early life factors: **A** 100 g increase in birth weight, unadjusted; **B** 100 g increase in birth weight (adjusted for gestational age and sex), **C** presence of prematurity; **D** birth weight to placenta weight ratio one unit increase, adjusted for gestational age and parity; y axis—estimated glomerular filtration ratio

Multivariable linear regression analysis (Table 2) showed that for every 100 g increase in birth weight (adjusted for gestational age and sex), the predicted eGFR *increased* by 0.3 mL/min/1.73 m^2^ (CKD-EPI cystatin C equation). The presence of prematurity is associated with predicted eGFR *decrease* by 3.23 mL/min/1.73 m^2^ (CKD-EPI cystatin C equation) over the mean follow-up time of 58 ± 5 years. Sex-specific analysis revealed that in women, for every 100 g increase in birth weight, the predicted eGFR *increased* by 1.00 mL/min/1.73 m^2^ (both CAPA and CKD-EPI cystatin C equations). Whilst in men, for every increase in 1 unit in birth weight to placenta weight ratio, the predicted eGFR *decreased* by 1.67 mL/min/1.73 m^2^ (LMrev equation). eGFR in this analysis was adjusted for age and pulse pressure (Table 2). The results of linear regression are visualized in Fig. 4.

**Table 2:** Early life factors as determinants of adult kidney function, measured by cystatin C or creatinine eGFR equations—following adjustments for age and pulse pressure

	*ß*	SE	*P*
All the subjects	CKD-EPI cystatin C		
Birth weight, g	0.002	0.001	0.015
Birth weight (adjusted for gestational age, sex), g	0.003	0.001	0.003
Prematurity, yes	− 3.23	1.589	0.043
	LMrev		
Birth weight to placenta weight ratio (adjusted for gestational age, parity)	− 1.99	0.82	0.015
WOMEN	CAPA		
Birth weight, g	0.001	0.002	0.045
	CKD-EPI cystatin C		
Birth weight, g	0.001	0.003	0.040
MEN	LMrev		
Birth weight to placenta weight ratio (adjusted for gestational age, parity)	− 1.67	0.89	0.045

Multivariable linear regression analysis

Multiple linear regression analysis results are also shown in Fig. 4

*CAPA* cystatin C eGFR equation based on Caucasian, Asian, Paediatric, and Adult cohorts, *LMrev* the Lund-Malmö revised creatinine based eGFR equation, *CKD-EPI cystatin C* the Chronic Kidney Disease Epidemiology Collaboration cystatin C equation, *CKD-EPI creatinine* the Chronic Kidney Disease Epidemiology Collaboration creatinine equation

### Risk of albuminuria

Logistic regression analysis showed that odds of albuminuria (adjusted for age and pulse pressure) was significantly *lower* in the subjects with higher either gender-specific or combined birth weight z-score (OR 0.78, 95% CI [0.61; 0.98]). This observation was specific for men (OR 0.75, 95% CI [0.58; 0.96]).

### Growth mismatch subgroup comparison: sex-specific differences

**Fig.5:**
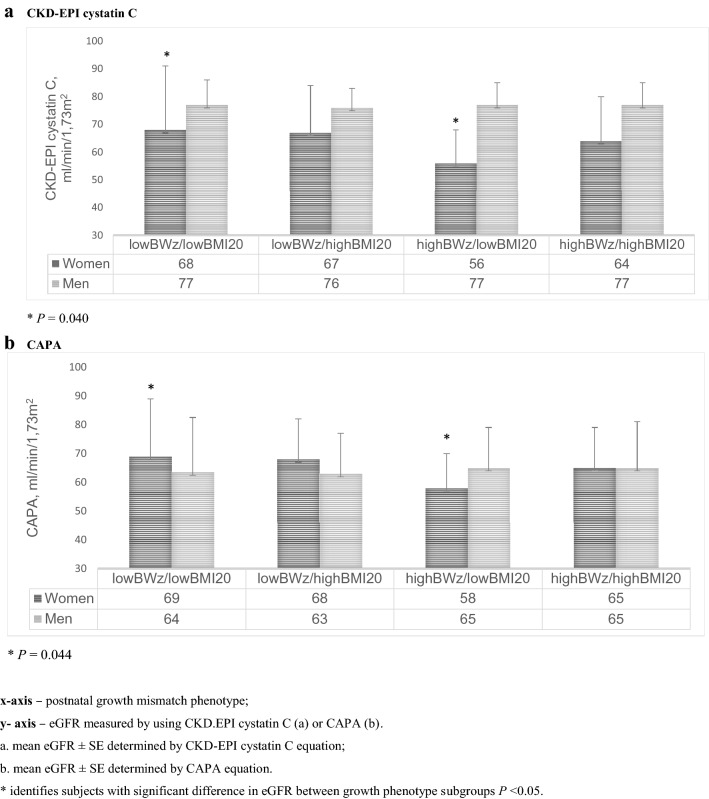
Post-natal growth patterns and adult kidney function as measured by cystatin C equations. **a** CKD-EPI cystatin C. *P = 0.040. **b** CAPA *P = 0.044. *x* axis—postnatal growth mismatch phenotype; *y* axis—eGFR measured by using CKD.EPI cystatin C (**a**) or CAPA (**b**). ^a^mean eGFR ± SE determined by CKD-EPI cystatin C equation; ^b^mean eGFR ± SE determined by CAPA equation. *Identifies subjects with significant difference in eGFR between growth phenotype subgroups *P* < 0.05

We found that women with *high-birth weight-z-score/lowBMI20* phenotype compared to *low-birth weight-z-score/lowBMI20 phenotype* (Fig. 5) had significantly *lower* eGFR adjusted for age and pulse pressure (*p* = 0.044 for CAPA, and *p* = 0.040 for CKD-EPI cystatin C). These associations were absent in men.

The odds of albuminuria (adjusted for age and pulse pressure) were significantly *lower* in men with *high-birth weight-z-score/highBMI20* phenotype compared to the reference group with low-birth weight-z-score/lowBMI20 (OR 0.35, 95% CI [0.12; 0.93]). The odds of albuminuria in other subgroups in men were as following: *low-birth weight-z-score/highBMI20*—OR 0.38, 95% CI [0.11; 1.09], *high-birth weight-z-score/lowBMI20*—OR 0.73, 95% CI [0.33; 1.64].

## Discussion

This observational, population-based study supports our hypothesis that in middle-aged and elderly Swedish subjects sex-specific associations exist between early life factors and kidney function, defined as cystatin C-based eGFR, as well as with albuminuria. This study also confirms that cystatin C compared to creatinine is a more sensitive marker for demonstrating these associations.

Despite the growing evidence that low birth weight together with developmental exposure to an adverse environment acting on the kidney might result in lower nephron/podocyte endowment [11, 34], there is still a critical need to identify early life factors that affect kidney function during the life course with the reporting of sex-stratified analyses.

Previous studies, including those listed in two recent meta-analyses, [7, 35] have mainly used creatinine-based equations for calculating adult eGFR with few exceptions of measured GFR [36] in women and cystatin C equation in a British population [37]. Moreover, the participants in these studies, except for two British reports [37, 38], were younger than 30 years old. Hence, most previous studies were based on plasma creatinine eGFR equations and had a significantly shorter follow-up time compared to our study. We show that subjects with lower birth weight adjusted for gestational age and sex, or those born preterm, are characterised by *lower* adult eGFR as determined by the CKD-EPI cystatin C equation, but not by eGFR creatinine equations. In women this association was even more pronounced, revealing a significant association between low birth weight and *low* eGFR acquired by both cystatin C equations (CAPA and CKD-EPI cystatin C).

Interestingly, we found that in men a decreased birth weight to placenta weight ratio, reflecting suboptimal fetal growth, when adjusted for age, gestational age, parity and pulse pressure, was related to *higher* eGFR based on creatinine (LMrev equation). In contrast, a decreased birth weight to placenta weight ratio has been previously reported to be related to worse perinatal outcome [39] and both neonatal and maternal morbidity [40]. This contradicts data from a Norwegian study [41] that showed an elevated risk for fetal death linked both to small and large placentas relative to birth weight in preterm births, and only in the group with small placentas relatively to birth weight at term. We propose that our finding may reveal those (male) individuals with compensatory glomerular hyperfiltration, a marker of both metabolic risk [42] and initial stages of CKD [43].

Furthermore, to the best of our knowledge we are the first to report that different postnatal growth mismatch phenotypes affect human adult kidney function. *Lower* cystatin C-based eGFR (both calculated using CAPA and CKD-EPI cystatin C equations) was observed in adult women with *high-birth weight-z-score/lowBMI20* phenotype indicating slowed postnatal growth. We could not find that in men. However, adult men with *high-birth weight-z-score/highBMI20* phenotype, indicating subjects with normal to high birth weight who had higher than median BMI at 20 years of age (> 21.7 kg/m^2^), had a *lower* risk of albuminuria. These findings highlight the importance of postnatal risk factors, e.g. over-nutrition [44], for the long-term kidney outcomes. Surprisingly, subjects born with low birth weight and the higher BMI gained during the first 20 years of life (the *mismatch* phenotype), did not have lower eGFR compared to the reference group. The *mismatch* phenotype has been previously linked to hypertension [45] and therefore the link to kidney function was expected in our cohort.

Albuminuria, an unfavourable marker of renal outcomes [46], has been previously linked to prematurity, low birth weight and fetal growth restriction [7, 18, 47]. Our findings were in accordance with reports showing *lower* risk of albuminuria in adults born with normal to higher birth weight, also evident in men. A 2008 report of the National Kidney Foundation's Kidney Early Evaluation Program [48] stated that low birth weight is related to CKD explicitly in young men at 18 years of age, but the authors did not elaborate on possible mechanisms. Besides, our failure to confirm further between early life factors and UACR could be related to other unmeasured confounders such as medications, measurement errors and urine sample collection variations.

Furthermore, our findings could be explained by several possible pathways. First of all, early life factors are associated with hypertension [12, 49] and could thereby indirectly cause kidney damage. Therefore, we adjusted our results for PP which particularly reflects arterial stiffness, a surrogate marker for vascular risk, and target organ damage [50, 51], as well as kidney function decline [52, 53]. Secondly, low nephron number at birth is assumed to directly cause premature kidney ageing and CKD [10]. Lastly, altered kidney function could be an epiphenomenon of maternal genetics for high blood pressure that has a secondary (indirect) effect on birth weight [54]. The findings of this study emphasize previous developmental research aimed at how early life factors affect future kidney damage that is essential for hypertension and ageing of the kidneys. The large reserve function of the kidneys has hampered detailed mechanistic understanding.

The strength of this study is that we report data after a long follow-up period, on average 58 years, which means current middle-aged and elderly Swedish subjects. Another strength is that we report kidney function measured by cystatin C and that we use different up-to-date cystatin C and creatinine eGFR equations against a background of different early life factors. Cystatin C is a more sensitive marker of kidney function compared to creatinine, as reported elsewhere [17]. It is well established that creatinine is muscle mass dependent and has lower sensitivity to detect early decreases in kidney function compared to cystatin C. Thus, our study, in contrast to studies with creatinine-based kidney function measurements, has the ability to detect associations earlier. We used both the common CKD-EPI equations, but also the LMrev and CAPA equations that were developed in the same general population from which the study cohort was retrieved in Sweden.

Limitations include that the study was restricted to people born in Sweden between 1926 and 1945, and that the study sample was relatively small. Our results could be biased due to the unequal sex distribution in the study sample, reflecting the source study (MDCS) [25], and the retrospective study design when adult participants of screening studies were back-tracked for birth data. One should not forget that there are metabolic factors, smoking, dysglycaemia, medication etc. that could have affected kidney function but were not included in this analysis that focused on early life influences.

*In summary*, here we report that a history of prematurity, irrespective of sex, and lower birth weight in women, are factors associated with *lower* cystatin C-based eGFR, while in men a history of lower birth weight (z score) is a risk marker for adult albuminuria. Cystatin C-derived eGFR equations show a higher ability to detect these findings compared to creatinine eGFR equations. A postnatal growth catch-up phenotype is not related to lower eGFR per se. The introduction of cystatin C in modern research has made it easier to describe these associations. We identified the protective phenotype (*high-birth weight-z-score/highBMI20*) for albuminuria in men and the unfavourable phenotype (*high-birth weight-z-score/lowBMI20*) for *lower* cystatin C-based eGFR in women. The observed association in men between birth weight to placenta weight and creatinine based eGFR (LMrev equation) could possibly indicate hyperfiltration and needs further investigation. The findings in general suggest that lower birth weight and postnatal growth phenotypes have a potential sex-specific influence on kidney function in middle-aged and elderly Swedish subjects. Our findings suggest that women seem to be more affected by early life factors when compared to men. Further studies are warranted to address the prognostic value of early life factors for adult kidney function and risk of renal disease across the life course.

## Data Availability

The datasets generated and/or analysed during the current study are available from the corresponding author on reasonable request.
